# Dimming the Powerhouse: Mitochondrial Dysfunction in the Liver and Skeletal Muscle of Intrauterine Growth Restricted Fetuses

**DOI:** 10.3389/fendo.2021.612888

**Published:** 2021-05-17

**Authors:** Alexander L. Pendleton, Stephanie R. Wesolowski, Timothy R. H. Regnault, Ronald M. Lynch, Sean W. Limesand

**Affiliations:** ^1^ School of Animal and Comparative Biomedical Sciences, The University of Arizona, Tucson, AZ, United States; ^2^ Department of Pediatrics, University of Colorado School of Medicine, Aurora, CO, United States; ^3^ Department of Physiology and Pharmacology, Western University, London, ON, Canada

**Keywords:** mitochondrial metabolism, tricarboxylic acid (TCA) cycle, intrauterine growth restriction (IUGR), placental insufficiency, oxidative phosphorylation

## Abstract

Intrauterine growth restriction (IUGR) of the fetus, resulting from placental insufficiency (PI), is characterized by low fetal oxygen and nutrient concentrations that stunt growth rates of metabolic organs. Numerous animal models of IUGR recapitulate pathophysiological conditions found in human fetuses with IUGR. These models provide insight into metabolic dysfunction in skeletal muscle and liver. For example, cellular energy production and metabolic rate are decreased in the skeletal muscle and liver of IUGR fetuses. These metabolic adaptations demonstrate that fundamental processes in mitochondria, such as substrate utilization and oxidative phosphorylation, are tempered in response to low oxygen and nutrient availability. As a central metabolic organelle, mitochondria coordinate cellular metabolism by coupling oxygen consumption to substrate utilization in concert with tissue energy demand and accretion. In IUGR fetuses, reducing mitochondrial metabolic capacity in response to nutrient restriction is advantageous to ensure fetal survival. If permanent, however, these adaptations may predispose IUGR fetuses toward metabolic diseases throughout life. Furthermore, these mitochondrial defects may underscore developmental programming that results in the sequela of metabolic pathologies. In this review, we examine how reduced nutrient availability in IUGR fetuses impacts skeletal muscle and liver substrate catabolism, and discuss how enzymatic processes governing mitochondrial function, such as the tricarboxylic acid cycle and electron transport chain, are regulated. Understanding how deficiencies in oxygen and substrate metabolism in response to placental restriction regulate skeletal muscle and liver metabolism is essential given the importance of these tissues in the development of later lifer metabolic dysfunction.

## Introduction

Many cases of intrauterine growth restriction (IUGR) of the fetus are caused by reductions in placental mass and function resulting in lower fetal nutrient and oxygen availability. Fetal development and growth are particularly vulnerable to perturbations in the *in utero* environment, and it is proposed that lower nutrient availability during gestation significantly impacts short- and long-term metabolic regulation (1–5). Glucose is an important and primary substrate for oxidative metabolism in the fetus (6, 7). Consequently, early studies in “small for gestational age” or IUGR neonates have focused on describing the negative effects from hypoglycemia and malnutrition on neonatal growth and metabolism (8–13). These studies laid the foundation for our understanding of neonatal metabolism and the potential responses associated with fetal growth restriction. However, there are gaps in our knowledge regarding how organ specific effects coordinate whole-body substrate utilization in the IUGR fetus.

Prenatal development establishes the foundation for global metabolic regulation and substrate utilization in fetal tissues. Glucose, lactate, and amino acids comprise the primary oxidizable substrates used for energy production and tissue growth in the fetus (14). When the placental transport capacity for nutrients and oxygen becomes limited in IUGR pregnancies, metabolic and endocrine responses are initiated in the fetus to ensure survival (15, 16). Moreover, as the fetal milieu changes, this subjects fetal tissues to hypoxia and hypoglycemia which promotes systemic responses such as increased oxidative stress (17, 18). As a result, two major physiological responses occur out of necessity: stunted growth and metabolic adaptation. Slowing growth lowers the global metabolic burden on the fetus and prioritizes substrate availability for essential organs such as the brain and heart. The skeletal muscle and liver each comprise a small amount of the total fetal weight near term (~10% for skeletal mass and 3–4% for liver mass) (19–22). However, combined, these tissues are responsible for 40–50% of the total fetal oxygen consumption, underscoring their importance as two of the largest metabolic organs in the fetus (19, 23). Therefore, these tissues are at greater risk for developing persisting metabolic adaptations when IUGR occurs.

IUGR neonates are born with lower muscle and liver masses (24–30). The deficiencies in lean mass are retained through adulthood, despite adequate nutrient availability after birth (24, 26, 27, 31–33). This observation is especially important considering that tissue growth is dictated by energy homeostasis, and this balance is governed by substrate utilization and oxidative phosphorylation (34–37). Interestingly, IUGR infants have higher rates of resting energy expenditure, measured within the first few weeks after delivery, and subsequently undergo increased body mass (catch-up growth) (38, 39). However, these neonates fail to adequately increase muscle mass, which potentiates subcutaneous and hepatic fat deposition, and represents a modified metabolic phenotype (34–37, 40–43). The greater adiposity may further serve as a comorbidity that exacerbates the metabolic strain throughout the life-course, including the predisposition to nonalcoholic fatty liver disease (42–47). The life-long metabolic strain induced by post-natal catch up growth manifests as an increased risk of developing impaired glucose tolerance, dyslipidemia, and hypertension (45, 48). Thus, individuals born IUGR are at risk of increased morbidities due to impaired substrate metabolism combined with lower lean mass and greater adiposity. These observations highlight that placental restriction negatively impacts prenatal metabolism, alters lean-to-fat mass ratios, which, through developmental programming mechanisms, negatively affects postnatal energy balance.

Comprehensive studies evaluating whole-body metabolism and the coordinated tissue-specific responses in IUGR fetuses are beginning to define the impacts of nutrient and oxygen restriction during gestation. However, despite the growing number of experimental models and studies investigating IUGR metabolism, much remains unknown. Given the importance and centrality of mitochondria to substrate utilization and energy balance, the metabolic aberrations observed in IUGR fetuses implies mitochondrial metabolism is adjusted to low nutrient and oxygen concentrations. In this review, we will discuss our current understanding of mitochondrial metabolism in the skeletal muscle and liver of IUGR fetuses and present gaps in the field.

## Maintenance of Oxidative Phosphorylation in Mitochondria

Oxidative metabolism is predicated on mitochondrial function. Within mitochondria, carbohydrate (pyruvate), amino acid, and fatty acid metabolism intersect in the tricarboxylic acid (TCA) cycle, which requires oxygen to produce the primary cellular energy currency: adenosine triphosphate (ATP). However, utilization of substrates within the mitochondrion is not equal. For example, some metabolites, like lysine and leucine, only enter the TCA cycle only as acetyl-CoA. Alternatively, other substrates, like valine and glutamate, can only enter as TCA cycle intermediates *via* specific carriers. The latter substrate utilization process, known as anaplerosis, is vital to mitochondrial metabolism as it replenishes TCA cycle intermediates. Oxidative phosphorylation is generally limited by the presentation of ADP to the mitochondrion (free ADP), and changes in the concentration of any one substrate affect the utilization of other substrates (49).

In the mitochondrion, ATP is synthesized from the oxidation of pyruvate, amino acids, and lipids. To be utilized, these substrates must be transported into the mitochondrion across the outer and inner mitochondrial membranes. Once inside the mitochondrial matrix, metabolic substrates can enter the TCA cycle as acetyl-CoA or as an intermediate and ultimately results in the production of both NADH and FADH_2_. These reducing equivalents produced from each pass of the TCA cycle are subsequently oxidized at the electron transport chain (ETC) to produce ATP. While acetyl-CoA is the primary substrate for the TCA cycle, the acetyl CoA oxidation capacity of the TCA cycle is dependent upon the availability of TCA cycle intermediates. Specifically, anaplerotic reactions are needed to replace TCA cycle intermediates that are lost through cataplerosis.

The net effects of catabolism and anabolism on energy production define mitochondrial function. The TCA cycle and ETC are intrinsically linked through the redox cycle of NADH and FADH_2_ (50, 51). Normally, the flow of electrons starts with the oxidation of NADH and FADH_2_ at Complexes I and II, respectively. Alternatively, electrons can enter the ETC, downstream of Complex I, *via* glycerol-3-phosphate dehydrogenase, electron-transferring flavoprotein, or dihydroorotate dehydrogenase (DHODH) (52). As electrons are transported through the ETC, protons move from the matrix to the intermembrane space, creating the proton motive force. The journey of electrons through the ETC ends with their terminal transfer onto oxygen at Complex IV (oxygen consumption). However, because the ETC is not perfectly coupled, electrons can escape the ETC, leading to reactive oxygen species (ROS) production. Simultaneously, the proton motive force is used by ATP synthase to catalyze ATP from ADP and inorganic phosphate (P_i_). The integrated nature of the ETC and TCA cycle means the rate of oxygen consumption in the mitochondria is coupled to the phosphorylation potential [log (ATP)/(ADP) (P_i_)], which means ATP production and utilization are coupled to oxygen consumption rate (OCR) (51, 53–58). As such, conditions that reduce the electron flux through the ETC will also reduce the production of ATP as well as the regeneration of NAD+/FADH due to reduced NADH/FADH_2_ oxidation (51, 55, 57). Consequently, both cytosolic and mitochondrial enzymes that rely on hydrogen carriers to function, including those in the TCA cycle, are impacted by perturbations in ETC function, which subsequently reduces oxygen consumption and energy production.

## Animal Models of IUGR With Metabolic Dysfunction

Measuring whole-body, tissue, and cellular metabolism requires invasive experiments. As a result, animal models that recapitulate human IUGR are needed to study the link placental insufficiency and the metabolic phenotype of IUGR fetuses. Similar to human IUGR, several animal models of IUGR have been developed that exhibit reductions in lean body mass, lower blood nutrient and/or oxygen concentrations, and develop metabolic syndromes later in life (59–63).

### Rats

Three well-defined rat models of IUGR show fetal and persistent postnatal metabolic consequences in skeletal muscle and hepatic tissue due to reduced nutrient availability during gestation. Gestational maternal caloric restriction (CR), gestational maternal low protein (LP), and uterine artery ligation (UAL) at embryonic day 19 (E19), each causes significant (10–50%) reductions in lean mass which persist into adulthood (31, 40, 60, 64–67). The persistent disparity in lean mass may serve as an IUGR trait that underscores metabolic defects in skeletal muscle and liver function leading to glucose intolerance, fatty liver disease, and altered amino acid metabolism in juveniles with IUGR (33, 59, 60, 68–72). In addition to low bodyweight, IUGR fetal rats possess defects in oxidative phosphorylation and nutrient signaling in both the skeletal muscle and liver (32, 33, 40, 64, 65, 70, 73–78). Therefore, although the exact mechanisms behind the development of metabolic dysfunction in IUGR rats are unclear, altered mitochondrial function acquired during fetal life is likely a contributing cause to the development of metabolic aberrations later in life.

### Sheep

Experimental models have been developed to induce IUGR in sheep by either reducing placental structure/function or preventing normal placental development. Models that reduce placental structure or function, such as carunclectomy or placental embolization, mimic placental insufficiency and yield fetuses with 15–66% growth restriction compared to matched controls (79–81). Models that inhibit placental development, such as maternal hyperthermia and over-nourished adolescent ewes, also produce IUGR of the fetus. Fetal weights in these sheep models of IUGR are reduced by 30–60% when measured at 0.9 gestation (82–85).

Factors that match closely with the gradual onset of IUGR found in human pregnancies are recapitulated in sheep models (86–88). Placental insufficiency-induced IUGR sheep fetuses have severe nutrient restriction in late gestation that results in hypoxemia, hypoglycemia, as well as reduced transport of amino acids (15, 89–92). To ensure survival, the IUGR sheep fetus copes with lower nutrient and oxygen availability by slowing skeletal muscle growth and increasing hepatic glucose production rates (93–95). These *in utero* adaptations lead to postnatal metabolic dysfunction where young lambs afflicted with IUGR continue to have lower lean masses and skeletal muscle protein contents, altered glucose metabolism and liver function, and greater adiposity (96–100). Considering the mitochondria are the primary site for oxidative metabolism, these adaptations imply IUGR fetuses have altered hepatic and skeletal muscle mitochondrial function.

### Piglets

Natural runting of piglets (IUGR) arises from a disproportionate supply of nutrients along the uterine horn causing growth restriction of 15–20% of piglets from each litter (101). As a result of *in utero* nutrient restriction, runted piglets exhibit asymmetric growth restriction, increased liver:brain weight ratio, reduced bodyweight at birth, and metabolic perturbations which last into adulthood (62, 102–104). Consistent with human IUGR pathophysiology, the lower lean mass present in IUGR piglets at birth is never fully rectified in adulthood (105, 106). IUGR piglets at term also have a lower capacity for skeletal muscle protein accretion and protein synthesis (107, 108). The impact of IUGR not only decreases liver and skeletal muscle growth, but also decreases the abundances of proteins involved in intermediate metabolism in the liver and energy production in the skeletal muscle (107). As adults, energy production, amino acid catabolism, and glucose metabolism remain dysregulated in both the liver and skeletal muscle (62, 109).

Clues to metabolic adaptation of IUGR piglets may be found in studies using nutrient supplementation. Specifically, mid-gestation arginine/glutamine supplementation of pregnant gilts simultaneously increases litter size and reduces the number of IUGR piglets per litter (108). Conversely, neonatal supplementation of amino acids to IUGR piglets results in hyperammonemia, elevated blood urea concentrations, and death (110). Moreover, during the first 21 days post-parturition, postnatal glucose injections increased IUGR piglet bodyweight compared to non-supplemented IUGR piglets (111). Based on these observations, IUGR piglets may have predetermined fuel preferences that are programmed *in utero*. Thus, in IUGR piglets, the asymmetric growth restriction and metabolic dysfunction indicate defects in mitochondrial function that continue postnatally (112–117).

## Mitochondrial Metabolism and Dysfunction in Animal Models of IUGR

The conservation and scavenging of oxygen and nutrients from expendable metabolic processes, such as systemic growth, are a necessary adaptation during IUGR to meet basal metabolic demands. Accordingly, because protein synthesis during the latter half of gestation accounts for ~18% of fetal oxidative metabolism, and protein synthesis is an ATP consuming process, slowing protein accretion may be a primary mechanism of conserving energy, oxygen, and nutrients in IUGR fetuses (118, 119). On the other hand, IUGR fetuses have an early activation of hepatic glucose production, an ATP and substrate demanding process that is absent in the normal fetus but activated to counteract hypoglycemia (7, 74, 120). These two observations show that differential metabolic adaptations are needed during IUGR. Further, these pathways likely have tissue-specific regulation that depends on differential enzyme expression in the skeletal muscle and liver. From this, a critical question arises: how are these metabolic processes regulated in skeletal muscle and the liver of IUGR fetus?

### Skeletal Muscle

**Figure 1:**
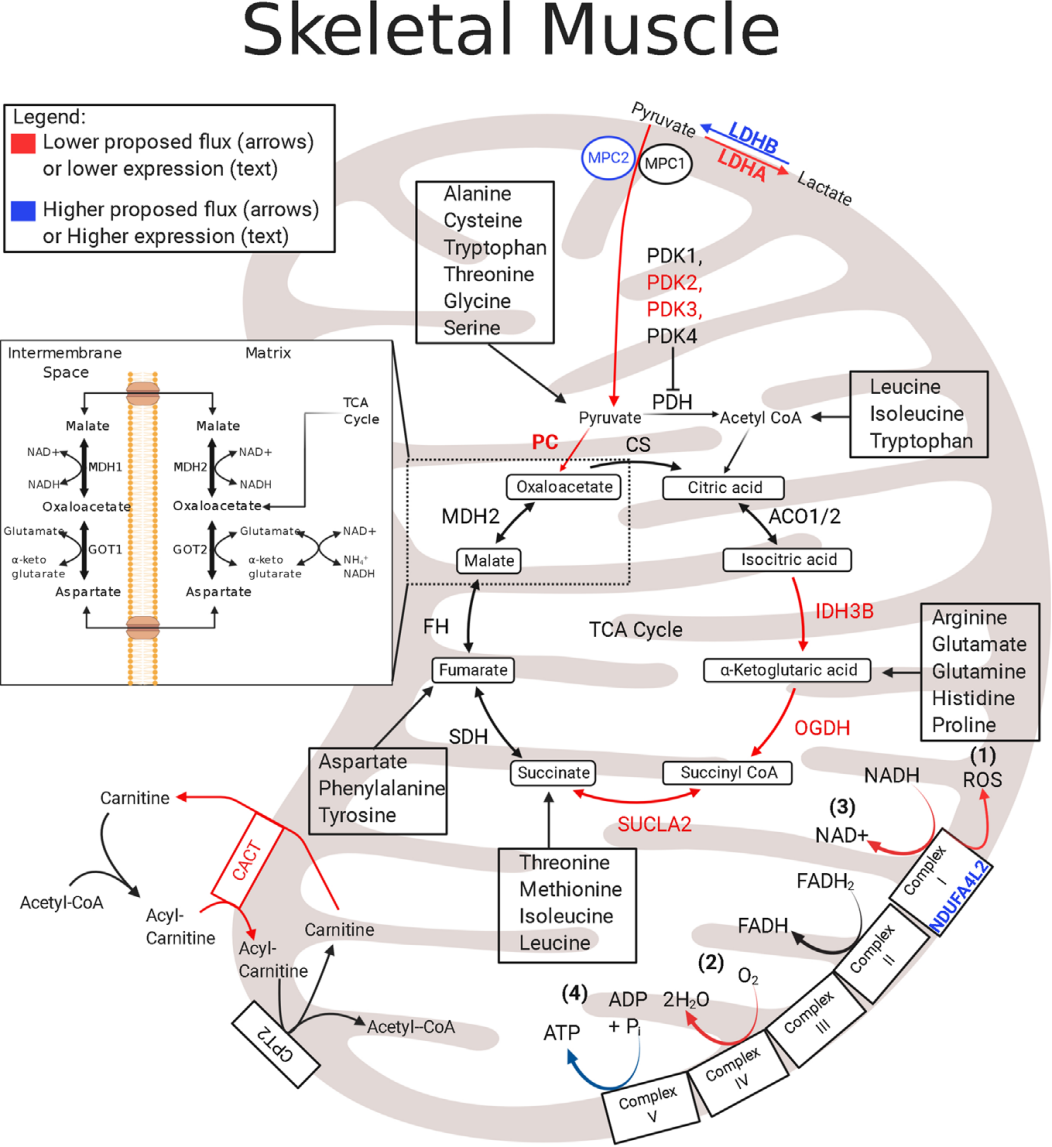
Enzymatic profile for skeletal muscle mitochondria in the IUGR fetus. The schematic outlines major mitochondrial enzymes and their processes in the tricarboxylic acid (TCA) cycle and in the electron transport chain (ETC). Lower abundances (red text) and greater abundances (blue text) for IUGR mitochondria are indicated, whereas enzymes that are not different from control mitochondria are in black text. Proposed decreased metabolic fluxes in IUGR mitochondria are shown with red (lower) and blue (higher). Here, fluxes of pyruvate, amino acids, and fatty acids are all thought to be lower in the IUGR skeletal muscle due to lower enzyme expression involved in each pathway. Initial pyruvate flux is thought to be hindered by higher MPC2 expression and is believed to be further slowed by lower expressions of IDH, OGDH, and SUCLA2. Because these enzymes are also innately involved in amino acid flux into the TCA cycle, amino acid degradation is also believed to be lower in IUGR skeletal muscle mitochondria. Moreover, higher NDUFA4L2 expression in IUGR skeletal muscle mitochondria is believed to slow electron flux leading to a) lower reactive oxygen species (ROS) production b) lower oxygen consumption rates c) lower NADH oxidation. ACO1/2^†^, aconitate hydratase 1/2 (121); CACT^†^, carnitine–acylcarnitine carrier (121); CPT2^†^, carnitine palmitoyltransferase 1,2 (121); CS^†^, citrate synthase (121); FH^†^, fumarate hydratase (121); GOT1, Glutamic-oxaloacetic transaminase 1; GOT2^†^, glutamic-oxaloacetic transaminase 2 (121); IDH^†^, isocitrate dehydrogenase (121); LDHA^‡^, lactate dehydrogenase A (121); LDHB^†^, lactate dehydrogenase B (121); MDH1, malate dehydrogenase 1 (121); MDH2^†^, malate dehydrogenase 2 (121); MPC2^†^, mitochondrial pyruvate carrier 2 (121); NDUFA4L2^†^, NDUFA4 mitochondrial complex associated like 2 (121); OGDH^†^, oxoglutarate dehydrogenase (121); PC^#^, pyruvate carboxylase (121, 124); PDH^●†^, pyruvate dehydrogenase (121); PDK1-4^#^, pyruvate dehydrogenase kinases 1-4 (85, 121); SDHA–D^†^, succinate dehydrogenase A–D (121); SUCLA2^†^, succinate-CoA ligase ADP-forming subunit *β* (121). ^†^denotes protein data, ^‡^denotes mRNA data, ^#^denotes protein and mRNA data. ^●^denotes activity data. The image was created in BioRender.com.

In fetal sheep with IUGR, reductions in skeletal muscle oxidative phosphorylation result from chronic reductions in both the ETC activity and TCA cycle protein abundances (121). A modulator of electron transfer to the ETC is NADH dehydrogenase 1 alpha subcomplex, 4-like 2 (NDUFA4L2) (122, 123). NDUFA4L2 is driven by hypoxia-inducible factor 1*α* (HIF1*α*), and it incorporates into Complex I and lowers the electron flux through the ETC by functioning as an inhibitor of oxidative phosphorylation (**Figure 1**) (121–123, 125). NDUFA4L2 expression is increased in the skeletal muscle of IUGR sheep fetuses, likely due to chronically sustained HIF1*α* activity, thereby exerting a global impact on suppressing whole-body metabolism (126–128). First, the lower electron flux through the ETC and complex 1 will lower the production of ROS, which will potentially protect against oxidative stress found in several pathologies, including IUGR, as a result of chronic hypoxia (**Figure 1**) (122, 129). Second, the lower electron flux through the ETC will reduce OCR as evidenced by the lower Complex I activity and state 3 (Complex I-mediated) flux observed in the skeletal muscle mitochondria of IUGR sheep fetuses (**Figure 1**) (121). Third, lower ETC electron flux and lower OCR will reduce NADH/FADH_2_ regeneration thereby inhibiting TCA cycle enzymes that use NAD+ as a cofactor (**Figure 1**). This is evidenced by lower abundances of key NAD+ consuming TCA cycle enzymes isocitrate dehydrogenase (IDH) and 2-oxo-glutarate dehydrogenase, also known as *α*-ketoglutarate dehydrogenase (OGDH) (**Figure 1**) (121). Fourth, and finally, concomitantly lowering NADH/FADH_2_ production and oxidation may place the skeletal muscle in a lower steady state by limiting energy (ATP) production and ADP rephosphorylation and may have significant consequences on basic cellular metabolic rates. The impact of NDUFA4L2 on whole-body metabolism is demonstrated in transgenic mice with NDUFA4L2 overexpression in the skeletal muscle (122). NDUFA4L2 reduces skeletal muscle mass by ~20%, mirroring the slow muscle growth in IUGR fetuses (122). These adjustments to mitochondrial function lowers the rates of ATP production in sync with other ATP-dependent processes, such as lower Na+/K+ ATPase activity and protein synthesis (130). Therefore, skeletal muscle in the IUGR fetus has lower ETC flux to match lower oxygen availability *via* increased NDUFA4L2, but ATP utilization is reduced to parallel production.

Reduced ETC flux and ATP production indicate that utilization of oxygen and metabolic substrates is curtailed. Hindlimb oxygen uptake (consumption) relative to mass is 29% lower in IUGR fetuses compared to that in controls, which is consistent with depressed oxidative metabolism (94). Despite reductions in substrate oxidation, metabolic quotients for individual substrates were examined to observe substrate preferences for oxidative metabolism (94). Here, substrate oxygen quotients were calculated by dividing the whole blood arterial–venous difference in substrate concentration by the arterial–venous difference in oxygen content, then multiplying by the number of oxygen molecules needed to oxidize one molecule of the nutrient (94, 131). Despite similar glucose + lactate oxygen quotients across the hindlimb of the IUGR fetal sheep, the glucose uptake and lactate output per mole of oxygen consumed by the hind limb are greater (94). Interestingly, the hindlimb amino acid oxygen quotient is significantly reduced in IUGR fetal sheep (94). When the sum of these oxidizable substrates is calculated, they modestly exceed the oxygen consumption rate (nutrient to oxygen quotient is approximately 1), meaning the substrates are meeting the minimal energy requirement of the muscle but are not substantially contributing to tissue accretion (94). The results from the fetal sheep hindlimb reflect findings in the whole fetus where lower fetal uptake of amino acids is the dominant factor limiting growth and is associated with hypoxemia (131). Although the hypoxic induction of NDUFA4L2 will lower oxygen consumption in the mitochondria, additional mechanisms are likely responsible for the reduction in amino acid uptake and oxidation (94, 122, 125, 131, 132). As a result, the skeletal muscle of IUGR fetuses is in a lower metabolic state that is fine-tuned towards maintenance rather than growth by matching substrate utilization to oxygen availability (94, 124).

### Liver

**Figure 2:**
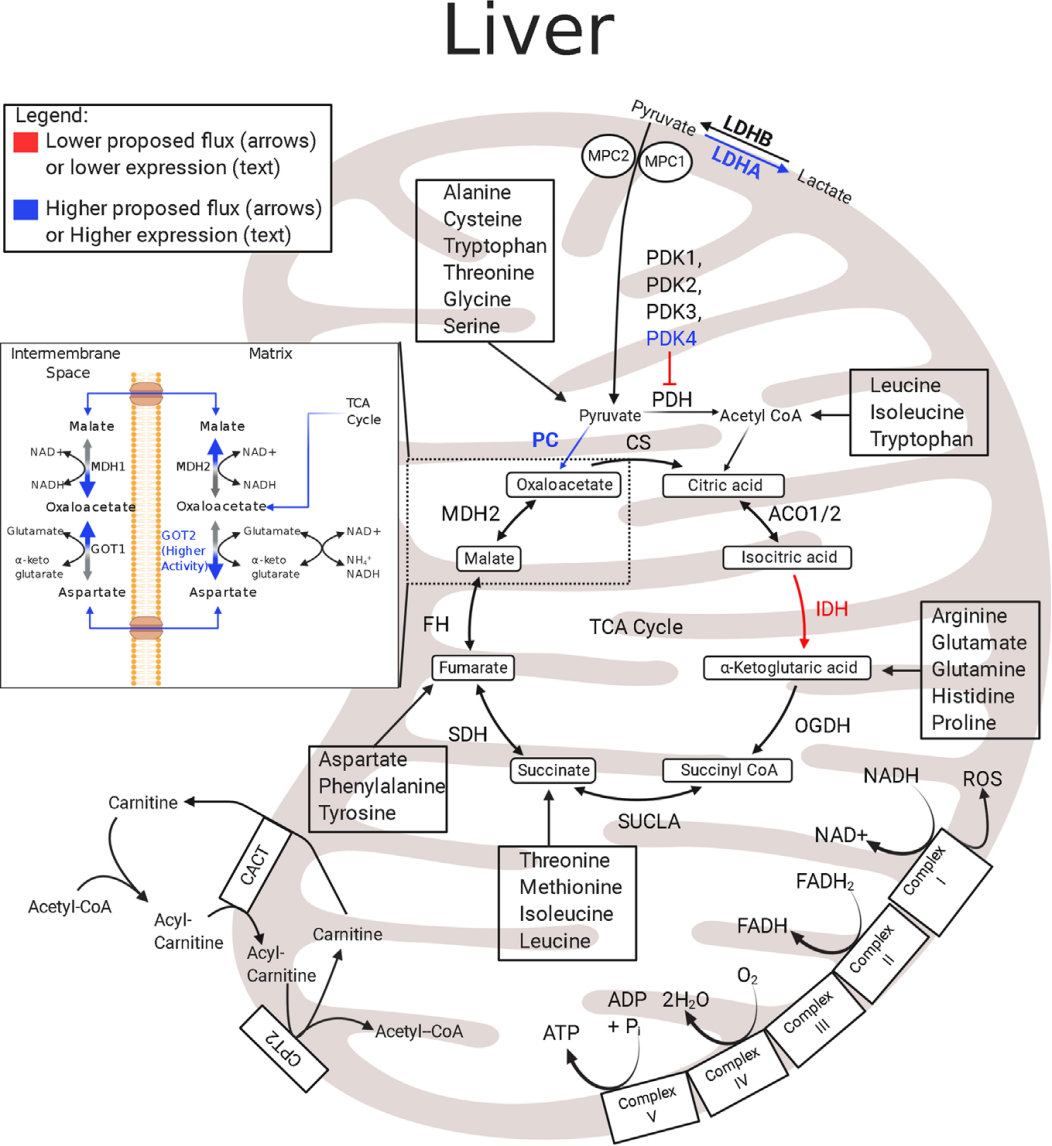
Enzymatic profile for liver mitochondria in the IUGR fetus. The schematic outlines major mitochondrial enzymes and their processes in the tricarboxylic acid (TCA) cycle and electron transport chain (ETC). Lower abundances (red text) and greater abundances (blue text) for IUGR mitochondria are indicated, whereas enzymes that are not different from the control mitochondria are in black text (133, 135, 136). Proposed decreased metabolic fluxes in IUGR mitochondria are shown with red (lower) and blue (higher) arrows. Here, pyruvate oxidation in the hepatic mitochondria is speculated to be lower due to higher PDK4 and LDHA expression. In addition to lowering pyruvate oxidation, these changes are thought to push pyruvate flux towards gluconeogenesis, evidenced by higher PC expression and lower IDH expression. The lower IDH expression may act to slow pyruvate oxidation in the hepatic TCA cycle upstream of *α*-ketoglutarate and allow amino acids to become the primary oxidizable substrate in the TCA cycle downstream of IDH. ACO1/2, aconitate hydratase 1/2; CACT, carnitine-acylcarnitine carrier; CPT2, carnitine palmitoyltransferase 1,2; CS, citrate synthase; FH, fumarate hydratase; GOT1, glutamic-oxaloacetic transaminase 1; GOT2^●^, glutamic-oxaloacetic transaminase 2 (136); IDH^‡^, isocitrate dehydrogenase (133); LDHA^‡^, lactate dehydrogenase A; LDHB, lactate dehydrogenase B; MDH1, malate dehydrogenase 1; MDH2, malate dehydrogenase 2; MPC2, mitochondrial pyruvate carrier 2; OGDH, oxoglutarate dehydrogenase; PC^‡^, pyruvate carboxylase (135); PDH^‡^, pyruvate dehydrogenase (135); PDK1-4^‡^, pyruvate dehydrogenase kinases 1-4 (135); SDHA–D, succinate dehydrogenase A–D; SUCLA2, succinate-CoA ligase ADP-forming subunit β. ^‡^denotes mRNA data, ^●^denotes activity data. The image was created in BioRender.com.

In the IUGR fetus, the liver also has low rates of growth, modified protein synthesis rates, and a low mitochondrial redox state coupled with low concentrations of TCA cycle intermediates (32, 107, 133, 134). Although IUGR sheep hepatocytes are postulated to have lower energy production, the abundances of the hepatic ETC and TCA cycle metabolites appear unaffected, and the expression levels of ETC enzymes are presently unknown (**Figure 2**) (133). Hepatic expression of IDH mRNA is lower in IUGR fetal sheep than in controls and may slow TCA cycle functionality upstream of *α*-ketoglutarate (133). These differences may reflect the dynamic role of the liver during placental restriction. The IUGR liver has an early activation of glucose production and thus must maintain energy requirements for gluconeogenesis. However, achieving this metabolic goal presents a unique problem to the IUGR liver when trying to conserve nutrients and energy utilization. Specifically, energy production relies on continuous revolutions of the TCA cycle to reduce cofactors thus maintenance of TCA cycle intermediates. However, the high rate of gluconeogenesis demands a high flux of oxaloacetate out of the TCA cycle, lowering the potential for energy production (ATP and NADH). Therefore, as oxaloacetate leaves the mitochondrial matrix, it must be replenished to retain the functionality of the TCA cycle. Pyruvate is the primary source for both energy production (*via* acetyl-CoA) and gluconeogenesis (*via* oxaloacetate), and increased lactate flux from the skeletal muscle likely counterbalances pyruvate deficits in the IUGR liver (93, 135). Even so, increased HGP likely requires additional TCA cycle intermediates for both the gluconeogenic substrates and the energy production requirements needed to sustain gluconeogenesis. This may include increased utilization and oxidation of amino acids to supply the carbon and energy substrates for gluconeogenesis, as demonstrated in studies with hypoglycemia-induced gluconeogenesis in the fetus, or increased intrahepatic lactate production and utilization (23, 135). The lower IDH mRNA abundance in the IUGR liver may act to redirect pyruvate flux towards gluconeogenesis, but allows downstream substrates, like amino acids, to enter the TCA cycle. Thus, in the IUGR fetus the liver may have an increased reliance upon anaplerotic substrates to replenish TCA cycle intermediates compared to the skeletal muscle.

## Pyruvate: Differential Regulation of Pyruvate Oxidation in IUGR Tissues

Glucose and lactate oxidation account for ~65% of oxygen consumption in the normally developing sheep fetus (7, 137). However, glucose metabolism in the skeletal muscle and liver is differentially regulated. In the fetus, the muscle is a major consumer of glucose, in contrast to the liver which is a small net consumer of glucose and is capable of producing glucose (6).

The IUGR fetus has tissue specific differences in insulin sensitivity for glucose metabolism (138). IUGR fetuses, studied under basal conditions, have normal or near normal rates of weight-specific, whole-body glucose utilization even with ~30% lower umbilical glucose uptake (6, 139). This reflects the early activation of endogenous glucose production to meet the fetal demand for glucose utilization (93, 140). Furthermore, at the whole-body level, IUGR fetuses have increased insulin sensitivity for glucose utilization given the normal utilization of glucose at lower insulin levels and higher utilization rates at high insulin doses when tested under hyperinsulinemic–euglycemic conditions to measure insulin sensitivity (120, 140). Lastly, the early activation of hepatic glucose production in IUGR fetuses is not suppressed by insulin, demonstrating the early development of liver-specific insulin resistance (120, 134, 139, 141–143). Together, these studies demonstrate that tissues like skeletal muscle are insulin-sensitive for glucose utilization, while the liver has developed insulin resistance and continues to produce glucose for the IUGR fetus (110, 138, 144).

The utilization of pyruvate, the oxidative product of glycolysis and product of lactate oxidation, has profound effects on energy status, redox state, and overall homeostasis. Pyruvate has two primary fates: entrance into the TCA cycle or conversion into lactate, which is a reversable reaction. For TCA cycle entry, pyruvate flux is regulated by pyruvate carboxylase (PC) and pyruvate dehydrogenase (PDH). PDH converts pyruvate to acetyl CoA, and PC is used for the anaplerotic “backfill” of oxaloacetate. Conversely, lactate dehydrogenase (LDH) facilitates the interconversion of pyruvate and lactate, both of which can be excreted. The pyruvate flux into the mitochondrion is regulated by PDH, and PDH activity is inhibited by its phosphorylation status *via* pyruvate dehydrogenase kinases (PDKs) and pyruvate dehydrogenase phosphatases (PDPs), which inactivate or activate PDH, respectively. In IUGR sheep fetuses, the skeletal muscle and liver glycolytic capacity appear normal or increased (85, 124, 135). However, because the skeletal muscle and liver have different metabolic roles in normal fetuses and during IUGR, adaptations in enzymes that regulate pyruvate metabolism are expected to differ (**Figures 1**, **2**) (85, 121, 135).

### Skeletal Muscle

Although the skeletal muscle of IUGR fetal sheep has three- to four-fold higher *PDK4* mRNA expression than controls, it has normal PDK4 protein abundance and higher PDH activity (85, 121, 135). Conversely, the protein and mRNA expression of *PC* is lower in the IUGR sheep skeletal muscle (121, 135). The lower *PC* expression and higher PDH activity in the skeletal muscle are conflicting as the lower expression of PC represents lower anaplerosis, and the higher activity of the PDH pathway represents enhanced pyruvate oxidation. However, this response may indicate, in part, that the minimal needs of the muscle can be met with pyruvate conversion to acetyl CoA making the IUGR skeletal muscle less reliant upon anaplerotic reactions through PC. These data may explain the disassociation between glucose utilization and oxidation discussed above.

Normal rates of pyruvate oxidation with greater PDH activity indicate deficiencies in pyruvate transport into the mitochondria of the IUGR skeletal muscle. The abundance of mitochondrial pyruvate carrier 2 (MPC2) is lower in the IUGR sheep skeletal muscle mitochondria (**Figure 1**) (121). Less MPC2 will restrict the transport of pyruvate into the mitochondria for PDH conversion. Due to the normal glycolytic capacity in IUGR sheep skeletal muscle, the inhibition of pyruvate flux into the mitochondrial matrix will lead to multiple metabolic outcomes (124). First, pyruvate will be converted to lactate prior to the TCA cycle, and this outcome is associated with greater intramuscular pyruvate concentrations and normal or increased plasma lactate concentrations in IUGR sheep fetuses (124). Second, by preventing pyruvate entry into the mitochondrial matrix, the matrix pyruvate concentrations will be lower, which will inhibit PDK4 activity, thereby leading to higher PDH activity, as evidenced by lower phosphorylation of PDH in the IUGR sheep skeletal muscle (85). Another fate for pyruvate may be conversion to alanine, consistent with increased Cahill cycling, and higher plasma alanine concentrations are observed in human and sheep IUGR fetuses (2, 124). Cahill cycling will keep skeletal muscle pyruvate oxidation low while also providing gluconeogenic substrates to the liver (133).

### Liver

In the fetal liver, under normal conditions, substrates are stored in the form of glycogen, used in protein synthesis, or used for energy production. Importantly, the fetal liver also normally has a high uptake of gluconeogenic substrates, yet it does not perform gluconeogenesis. The largest carbon sources for the fetal liver come from amino acids and lactate, with lesser contribution from glucose and a net output of pyruvate (23, 145, 146). However, under abnormal conditions, such as IUGR, increased gluconeogenesis is required to subsidize placental deficits in glucose supply to the fetus. While the carbon sources for increased gluconeogenesis are not known, if the IUGR fetus used pyruvate or lactate, this would necessitate an increased pyruvate to glucose conversion by modulating the abundances of enzymes that govern hepatic pyruvate metabolism to support gluconeogenic flux (**Figure 2**). In support of this, the IUGR sheep liver has increased PFK1 (phosphofructokinase 1), PDK4, and PC mRNA expression when compared to controls, along with increased expression of gluconeogenic genes, Phosphoenolpyruvate carboxykinase 1 and 2 (PCK1 and PCK2) (135). Curiously, IUGR fetuses also have increased *LDHA* mRNA expression which is suspected to increase intrahepatic lactate production (135). Taken together, these data suggest that increased PDK4 inhibits PDH and, with increased PC, favors conversion of pyruvate to oxaloacetate. Oxaloacetate is shuttled to the cytosol where PCK1 catalyzes the rate limiting step in gluconeogenesis and the resulting phosphoenolpyruvate (PEP) is used to synthesize glucose. Increased PCK2 also may catalyze the mitochondrial conversion of oxaloacetate to PEP. Additionally, increased LDHA may be important to produce lactate and regenerate NAD+ to sustain increased glycolysis *via* predicted increased PFK1 activity and maintain redox balance. Overall, these adaptations would permit a shift in substrate metabolism and energy production to support glucose production.

### Liver–Muscle Crosstalk

Evidence supports tissue-specific responses in the liver and skeletal muscle metabolism and crosstalk between these organs. Insight into interdependent metabolic regulation is realized from experiments on genetically engineered mice utilizing a skeletal muscle knockout (skmKO) of the MPC1 gene. Here, utilizing a skmKO of MPC1 reduces pyruvate flux through the MPC complex because MPC1 and MPC2 are both necessary for the functional MPC (147). Moreover, a knockout of MPC1 leading to a dysfunctional MPC may result in an increased reliance upon pyruvate–alanine cycling for forward TCA cycle flux, rather than anaplerotic filling *via* PC, as well as adaptive glutaminolysis (148). The former parallels the enzyme profile and predictions for alanine production in IUGR skeletal muscle, whereas the latter pathway is not expected in the IUGR muscle for reasons presented below (**Figure 1**) (148). Furthermore, this knockout model exhibits greater skeletal muscle lactate production, hepatic glucose production, and peripheral glucose disposal, all of which are found in the IUGR fetus (148). MPC1^skm−/−^ mice also have greater skeletal muscle fatty acid oxidation which acts to supplement acetyl CoA and succinate; this adaptation is likely specific to adult—rather than fetal—physiology (148). Due to lower rates of fatty acid oxidation in fetal tissues, the same metabolic flexibility observed in adult skeletal muscle is not likely to exist in IUGR fetuses. Nonetheless, the MPC1 mutation in the skeletal muscle enhances Cori and Cahill Cycle activity and indicates an intricate level of metabolic regulation between the skeletal muscle and liver that may be representative of aspects found in IUGR metabolism.

## Amino Acids: Divergent Metabolic Responses in Skeletal Muscle and Liver of the IUGR Fetus

The metabolism of amino acids supports oxidative phosphorylation and gluconeogenesis through reactions in the mitochondrion. Within the mitochondrial matrix, all 20 standard, proteinogenic amino acids (except cysteine, alanine, and histidine) have metabolic pathways which enter the TCA cycle. Though cysteine, alanine, and histidine do not enter the matrix, they can be converted into pyruvate or other amino acids, which can then be catabolized in the TCA cycle. In normally developing fetuses, amino acids are critical oxidative substrates, and the accretion of amino acids into proteins is an essential component for fetal growth. A common, overlapping feature between human IUGR and animal models of IUGR is reduced placental transfer of certain essential amino acids (134, 149–152). Although amino acids are critical substrates, specific mitochondrial amino acid metabolism in the skeletal muscle and liver of IUGR fetuses is largely unexplored. Furthermore, tissue-specific differences for amino acid utilization are expected in the IUGR fetus.

### Skeletal Muscle

In the skeletal muscle of IUGR sheep fetuses, substrate oxidation is modified through lower isocitrate dehydrogenase 3B (IDH3B), Succinate CoA ligase (SUCLA2), and OGDH abundance (**Figure 1**). Within the TCA cycle, IDH and OGDH act as rate limiting enzymes that regulate the rate of oxidation to meet the energy needs of the cell. Moreover, OGDH and SUCLA2 act as key anaplerotic gateways controlling much of the amino acid flux into the TCA cycle through the oxidation of *α*-ketoglutarate and succinyl-CoA, respectively. Therefore, downregulating the abundances of all of these enzymes establishes a complex substrate regulating system: the oxidation of upstream TCA cycle intermediates, including acetyl CoA, is inhibited by low IDH and OGDH abundances, and the anaplerotic flux of amino acids into downstream TCA cycle intermediates is inhibited by low OGDH and SUCLA2 abundances. In addition to the lower IDH, OGDH, and SUCLA2 concentrations, abundances of enzymes involved in branched-chain amino acid (BCAA) catabolism are lower, which will further reduce TCA cycle activity as BCAAs enter as acetyl CoA (leucine and isoleucine) or succinyl CoA (isoleucine and valine). Not only is TCA cycle activity depressed in IUGR fetal skeletal muscle, but it also appears to be less reliant upon amino acid substrates (94, 124)

**Figure 3:**
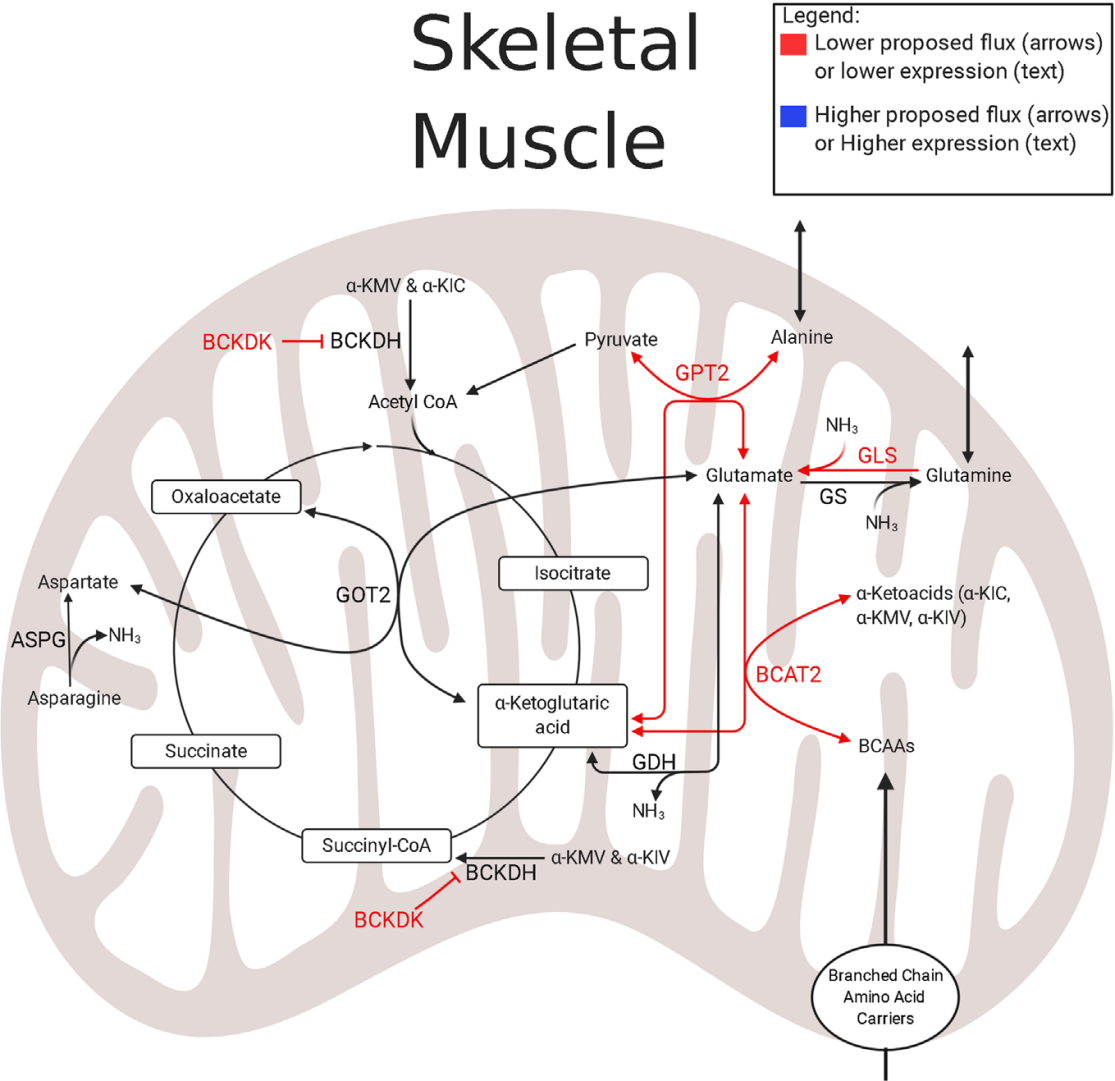
Amino acid and nitrogen-balance enzymes in IUGR skeletal muscle. The schematic outlines major mitochondrial enzymes and their processes in amino acid metabolism and nitrogen balance, created using BioRender.com. Lower abundances (red text) and greater abundances (blue text) are indicated for IUGR mitochondria compared to control mitochondria, whereas enzymes that are not different are in black text (109, 121, 124). Proposed decreased metabolic fluxes in IUGR mitochondria are shown with red (lower) and blue (higher) arrows. Here, amino acid flux into the TCA cycle through *α*-ketoglutarate may be lower due to lower expression of enzymes governing glutamate and BCAA degradation. These alterations in expression may allow the flux of ammoniagenic amino acids, such as glutamine and alanine, out of the skeletal muscle and into the blood stream. ASPG, asparaginase; BCAT2^#^, branched chain amino acid transaminase 2 (121, 124); BCKDK^†^, branched chain keto acid dehydrogenase kinase (121); GDH^†^, glutamate dehydrogenase (121); GLS^‡^, glutaminase (124); GOT2^†^, aspartate aminotransferase (121); GPT2^†^, alanine aminotransferase 2 (121); GS^#^, glutamine synthetase (121, 124). ^†^denotes protein data, ^‡^denotes mRNA data, ^#^denotes protein and mRNA data. The image was created in BioRender.com.

IUGR sheep fetal hindlimbs have lower net uptake rates of a variety of amino acids such as BCAAs, alanine, glycine, and glutamine (124). Coupled with the observation of lower abundances of mitochondrial enzymes associated with amino acid metabolism, amino acid oxidation is generally lower in IUGR skeletal muscle (94). In the muscle mitochondria of the IUGR sheep fetus, the first step of BCAA metabolism appears to be hindered by low expression of branched-chain aminotransferase 2 (BCAT2) (121, 124) (**Figure 3**). Conversely, the expression of the negative regulator, Branched Chain Keto Acid Dehydrogenase Kinase (BCKDK), is either lower or unchanged in both rat and sheep IUGR models (65, 121). These observations are conflicting, but clues to why these adaptations are advantageous to the IUGR skeletal muscle may lie within nitrogen disposal (**Figure 3**). In IUGR sheep fetuses, the abundance of glutamate–pyruvate transaminase 2 (GPT2) is lower in the skeletal muscle mitochondria, but the abundances of glutamate dehydrogenase (GDH), glutamate synthetase (GLS), and aspartate amino transferase (GOT2) are all unchanged (121, 124). Together, these alterations to enzyme abundances may represent an adaptation by IUGR skeletal muscle to increase nitrogen disposal and increase the efflux of gluconeogenic amino acids to the liver as they are all linked through the use of *α*-ketoglutarate and glutamate (124). Unfortunately, on its own, this enzyme profile is insufficient to decipher substrate flux, which identifies critical gaps for future studies to elucidate the amino acid metabolism in IUGR skeletal muscle.

### Liver

**Figure 4:**
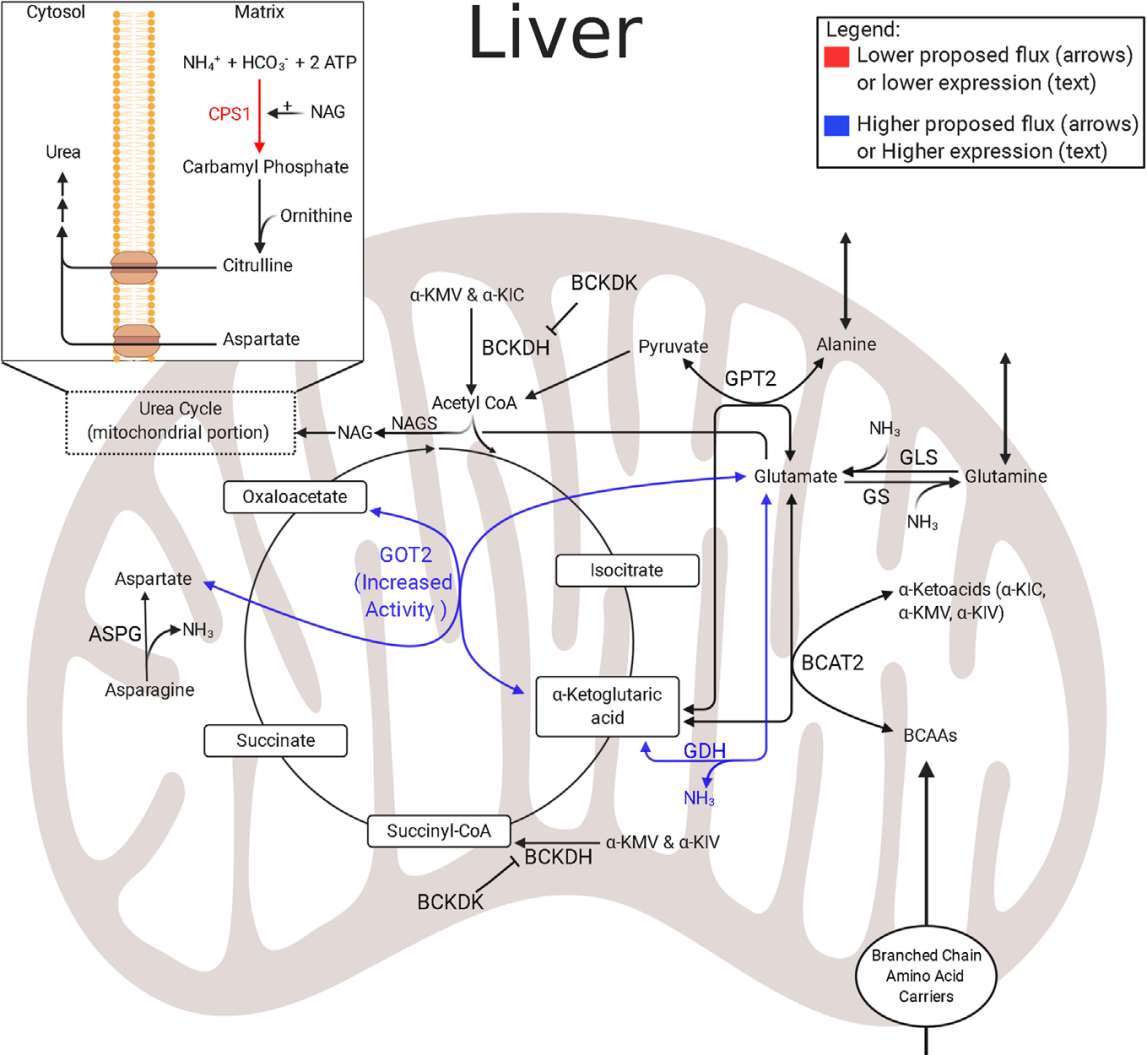
Amino acid and nitrogen-balance enzymes in IUGR liver. The schematic outlines major mitochondrial enzymes and their processes in amino acid metabolism and nitrogen balance, created using BioRender.com. Lower abundances (red text) and greater abundances (blue text) are indicated for IUGR mitochondria compared to control mitochondria, whereas enzymes that are not different are in black text (136). Proposed decreased metabolic fluxes in IUGR mitochondria are shown with red (lower) and blue (higher) arrows. The flux of glutamate into the hepatic TCA cycle is proposed to be higher due to higher expression or activity of enzymes governing glutamate metabolism, such as GOT2 and GDH. However, the suspected increased reliance on amino acids is thought to lead to increased ammonia production in the IUGR liver. This may be further complicated by an inability to release ammonia as urea due to lower CPS1 expression. ASPG, asparaginase; BCAT2, branched chain amino acid transaminase 2; BCKDK, branched chain keto acid dehydrogenase kinase; CPS1^†^, carbamoyl-phosphate synthase 1 (136); GDH^†^, glutamate dehydrogenase (136); GLS, glutaminase; GOT2^†^, aspartate aminotransferase (136); GPT2, alanine aminotransferase 2 (121); GS, glutamine synthetase; NAGS, N-acetylglutamate synthase. ^†^denotes protein data. The image was created in BioRender.com.

In normally growing fetuses, the hepatic uptake of essential and gluconeogenic amino acids is high (145, 146). Though amino acids are necessary to fetal metabolism, studies in fetal lambs and neonatal piglets show lower amino acid degradation and lower mitochondrial activity in the IUGR liver (133, 153). Further metabolomic studies of hepatic amino acid utilization in IUGR sheep fetuses show higher concentrations of BCAAs and alanine, while aspartate concentrations are lower (133). Interestingly, although amino acid degradation is lower in the IUGR liver, the lower amino acid degradation was primarily associated with arginine, histidine, proline, and tryptophan metabolic pathways—the first three pathways converge exclusively at *α*-ketoglutarate and indicate lower anaplerotic flux through *α*-ketoglutarate (**Figure 4**) (65). Furthermore, lower aspartate concentrations observed in the IUGR liver are indicative of adaptations which may be intended to mitigate high nitrogen concentrations that result from increased hepatic amino acid oxidation. Specifically, the IUGR skeletal muscle continually provides the liver with gluconeogenic amino acids, such as alanine and glutamine; a reaction that rids itself of ammonium while providing substrates to the liver for gluconeogenesis (124).

As the liver uses amino acids as energy substrates, they are deaminated, and ammonia is produced as a byproduct. Ammonia is toxic and is therefore typically excreted from the liver as innocuous urea. A common complication of being born IUGR is hyperammonemia (154, 155). This indicates that increased deamination of amino acids may persist due to lower abundances of enzymes responsible for the preceding steps of hepatic urea production, such as carbamoyl-phosphate synthase 1 (CPS1), in the mitochondria (**Figure 4**) (136, 154–156). This presents a difficult situation for the IUGR liver because the costs associated with hepatic gluconeogenesis cause the liver to become inundated with ammonia. Therefore, additional mechanisms are needed to manage excess hepatic ammonia concentrations. One relevant pathway to manage nitrogen balance is the malate–aspartate shuttle, which is also a primary energy shuttle between the mitochondria and cytosol (**Figure 2**). However, further studies are needed to resolve the mechanisms underlying hyperammonemia present in IUGR fetuses.

Gluconeogenesis relies on oxaloacetate as a substrate. However, the mitochondrial membrane is impermeable to oxaloacetate, and oxaloacetate must be converted to either malate or aspartate for transport (**Figure 2**). Although commonly grouped together as the “malate–aspartate shuttle,” each component has a distinct function. The malate shuttle requires a reducing step to carry oxaloacetate and electrons in the form of malate. Conversely, the aspartate shuttle relies on the availability of glutamate and *α*-ketoglutarate to carry oxaloacetate and nitrogen in the form of aspartate. These distinctions are important as the proportion of oxaloacetate carried by each of the shuttles additionally depends on the cytosolic redox state (NADH : NAD+ ratio) as well as the mitochondrial redox state. Moreover, the source of gluconeogenic fuels impacts which shuttle is used. If pyruvate is derived from lactate, the cytosolic NADH/NAD+ ratio will remain stable or increased, and the need for cytosolic reducing equivalents will be low, so oxaloacetate transport *via* the aspartate shuttle will predominate. Conversely, if pyruvate is derived from amino acids, no cytosolic reducing equivalents are produced. However, because gluconeogenesis requires glyceraldehyde-phosphate dehydrogenase, and this enzyme requires NADH, transport of oxaloacetate *via* the malate shuttle is necessary.

In IUGR sheep fetuses, the proportions of lactate and amino acids used for hepatic gluconeogenesis is unknown (133, 134, 138, 142). Studies investigating liver bioenergetics in IUGR rat fetuses have found discordant cytosolic and mitochondrial redox states, indicated by an increased cytosolic NAD+/NADH ratio, but a lower mitochondrial NAD+/NADH ratio (31, 32). Lower cytosolic, but higher mitochondrial, redox states suggest the malate shuttle is the preferred system used by IUGR mitochondria to produce cytosolic reducing equivalents and sustain gluconeogenesis. However, the aspartate shuttle may also be used to rid the IUGR hepatic mitochondria of excess ammonia (133). Additional investigations on mitochondrial metabolomics and proteomics are required to define adaptations in liver mitochondrial function.

## Lipids: A Minor Role in Fetal Metabolism

Compared to other metabolites discussed lipid metabolism remains relatively unexplored in both normally grown and IUGR fetuses. During pregnancy, maternal triglyceride (TAG) and free fatty acid (FFA) concentrations in the plasma range from 0.2 to 0.3 M for rats, sheep, and humans (157). Fetal plasma concentrations of FFAs are substantially lower than maternal concentrations (157). The concentration gradient might reflect low rates of placental transfer although small FFAs are able to cross the human placenta (158). However, fetal uptakes of FFA represent <10% of the total daily umbilical carbon uptake in both sheep and human fetuses (14, 159). Predictably, the FFA oxidation rate in the fetus is relatively low indicating FFA are incorporated into membranes or stored in the liver (160, 161). Despite low lipid oxidation rates, based on the relative contribution to the fetal metabolic rate (oxygen consumption), a small contribution to energy production may potentially affect the IUGR fetal outcomes. Interestingly, hepatic lipid content in IUGR fetal sheep is similar to controls (133), yet some studies demonstrate increased hepatic lipid accumulation in SGA or preterm human neonates (42, 43). Furthermore, the expression of lipid synthesis enzymes are lower in IUGR sheep fetuses suggesting that any lipids stored in the liver are exogenous and are supplied from the placenta (133). Conversely, maternal nutrient restriction in pregnant ewes leads to greater intramuscular triglyceride (IMTG) stores of the fetus (162). These observations imply that lipid oxidation, although already low in normal fetuses, is further reduced in IUGR fetuses. Moreover, the IUGR skeletal muscle and liver have different mechanisms to reduce lipid oxidation. In the skeletal muscle, IUGR sheep fetuses have a lower abundance of Carnitine-acylcarnitine translocase (CACT), which is responsible for the transport of carnitine and carnitine-fatty acid complexes across the inner mitochondrial membrane (**Figure 1**) (121). Low CACT concentrations would promote acetyl CoA oxidation from pyruvate or amino acids rather than FFAs. Therefore, lipid oxidation is likely regulated in a tissue-specific manner in IUGR fetuses.

## Conclusion

**Figure 5:**
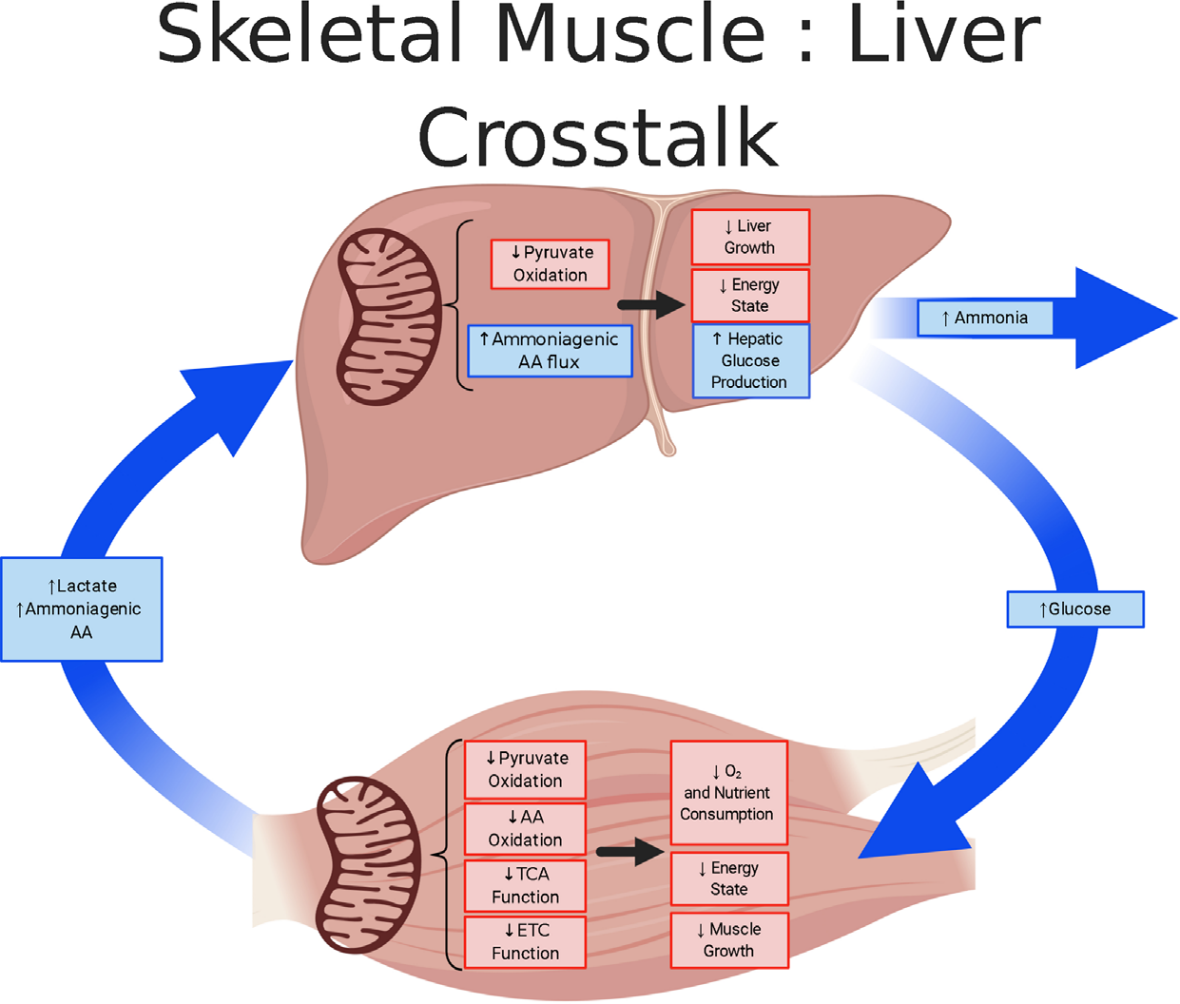
Skeletal muscle and liver metabolic cross-talk in IUGR fetuses. The schematic outlines the tissue metabolism outcomes due to mitochondrial adaptation in the skeletal muscle and liver of IUGR fetuses, created using BioRender.com. Lower responses (red) and greater responses (blue) are shown for IUGR mitochondria and whole tissue. The IUGR skeletal muscle is thought to be in a low energy, slow growing state in which substrate oxidation rates are lower. The adaptation by the IUGR skeletal muscle is thought to conserve oxygen, but also increase Cori (lactate) and Cahill (alanine) cycling. Conversely, the IUGR liver is proposed to use pyruvate supplemented with ammoniagenic amino acids to sustain increased glucose production. However, in the process of ammoniagenic amino acid degradation, this may lead to increased release of ammonia.

Animal models of IUGR provide valuable insight into the developmental origins of metabolic dysfunction. These models, while diverse in their etiology of IUGR, provide considerable evidence of irregularities in mitochondrial function. Skeletal muscle and liver serve as major metabolic tissues but exhibit distinct metabolic responses to fetal conditions causing IUGR (**Figure 5**). In the skeletal muscle of the IUGR fetus, the adaptations culminate in the creation of a “thrifty phenotype” that is slow growing with a low-energy demand. Phenotypic changes in IUGR muscle indicate that rates of glucose utilization and oxidation are disjointed and that the metabolic rate, measured with hindlimb oxygen consumption, is depressed. The greater utilization rates were hypothesized to promote anaerobic energy production *via* glycolysis (135). However as presented in this review, mitochondrial dysfunction is a primary site for metabolic dysregulation and programming which underlies a global physiological response in IUGR fetuses. Specifically, in IUGR skeletal muscle there is evidence for reduced TCA and ETC activity. Evidence for lower TCA is founded on reduced abundances of several enzymes and MPC2, whereas the ETC inhibition of Complex I activity result from the induction of the hypoxic-induced NDUFA4L2 inhibition. In contrast, to meet the increased energy demands associated with hepatic glucose production, the IUGR liver must utilize gluconeogenic substrates, likely a combination of pyruvate and amino acids, to provide the carbon substrates and energy co-factors to fuel gluconeogenesis. The mitochondrial metabolic adaptations of the skeletal muscle and liver and the modified crosstalk between these organs are important for minimizing energy demands in the IUGR fetus. However, as global metabolic aberrations develop in the fetus, they promote persistent adaptations that are deleterious throughout the individual’s life course.

## Author Contributions

AP was responsible for analysis and interpretation of data, drafting of manuscript, and critical revision. TR, SW, RL and SL were responsible for the interpretation of data and critical revision. All authors contributed to the article and approved the submitted version.

## Funding

NIH RO1 DK084842, NIH T32 HL007249, & R01-DK108910 supported this work.

## Conflict of Interest

The authors declare that the research was conducted in the absence of any commercial or financial relationships that could be construed as a potential conflict of interest.
